# Supplementary figures and images for: Evaluation of QuantiFERON SARS-CoV-2 interferon-γ release assay following SARS-CoV-2 infection and vaccination
Source: Clin Exp Immunol. 2023 Feb 21;212(3):249–61. doi: 10.1093/cei/uxad027 (PMC10243914; doi:10.1093/cei/uxad027)

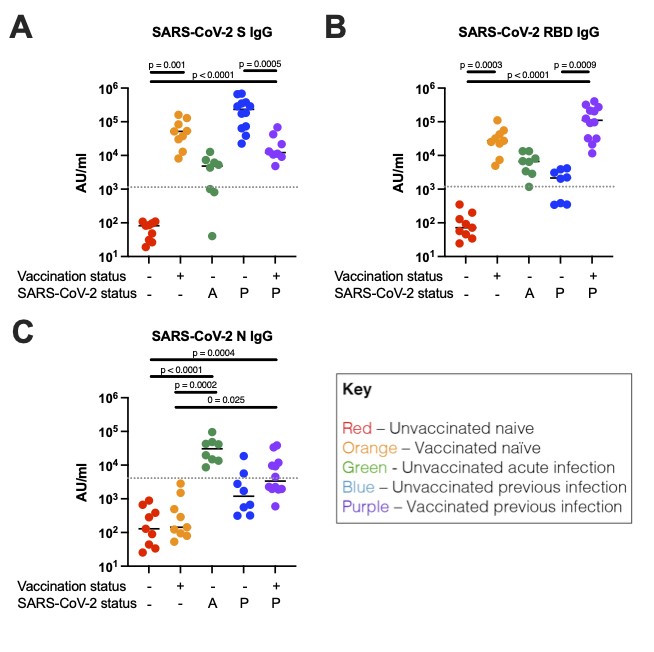

Supplement: uxad027_suppl_Supplementary_Figure_S1 [file uxad027_suppl_supplementary_figure_s1.jpeg]

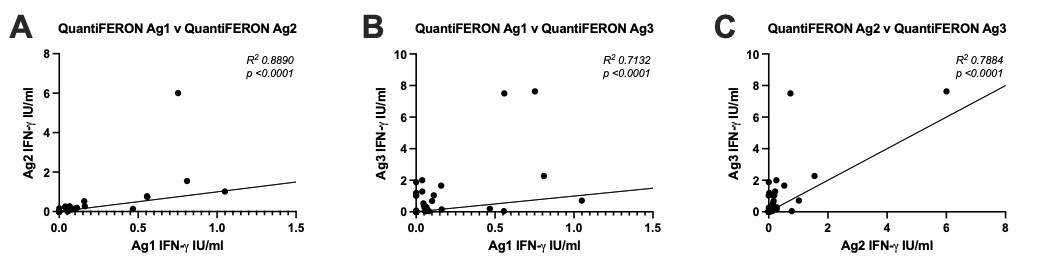

Supplement: uxad027_suppl_Supplementary_Figure_S2 [file uxad027_suppl_supplementary_figure_s2.jpeg]

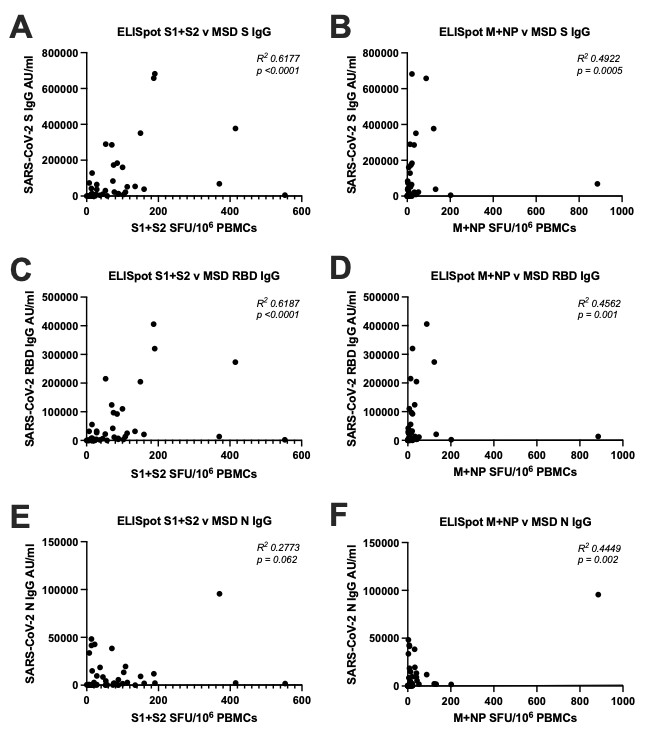

Supplement: uxad027_suppl_Supplementary_Figure_S3 [file uxad027_suppl_supplementary_figure_s3.jpeg]

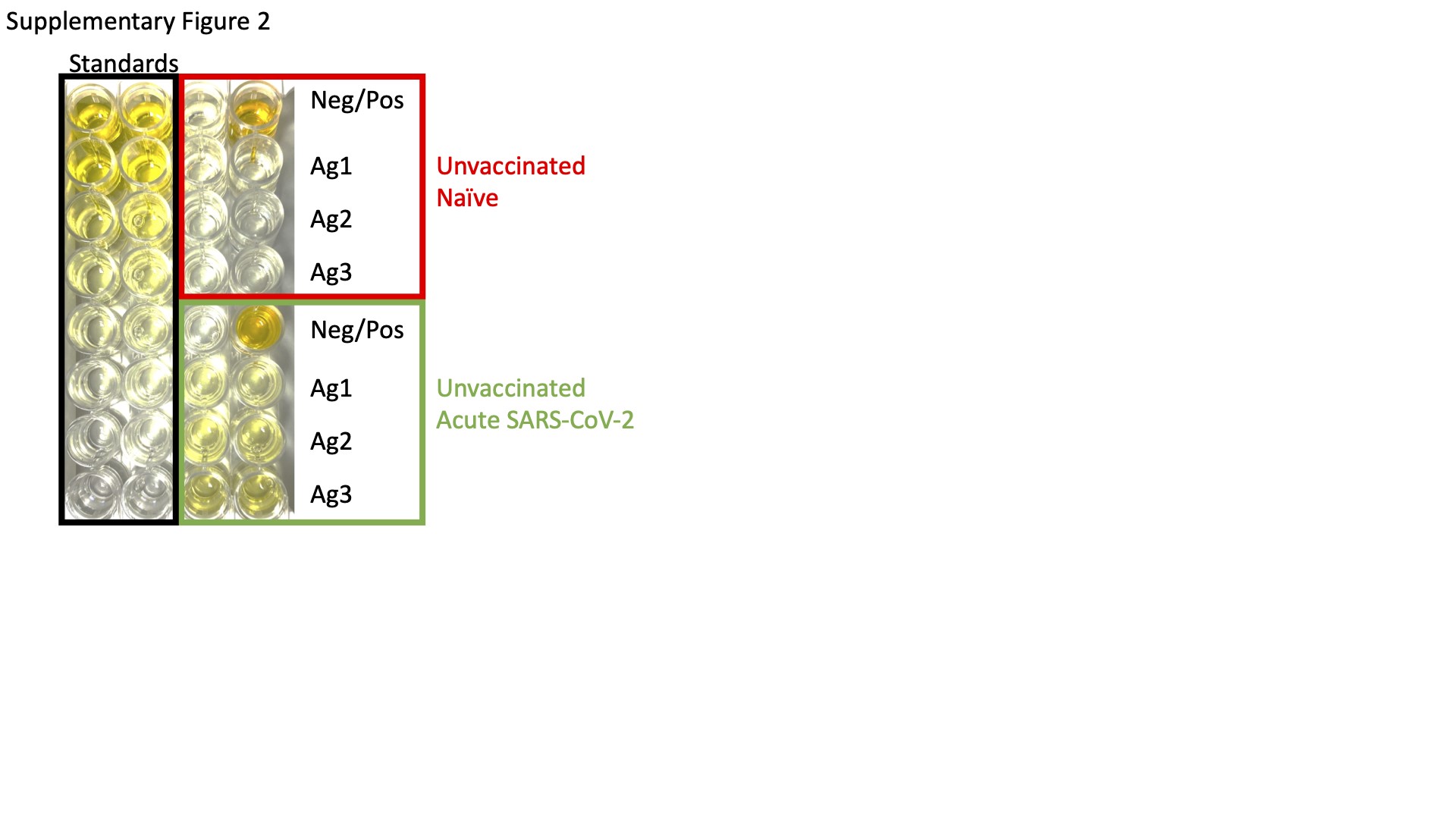

Supplement: uxad027_suppl_Supplementary_Figure_S4 [file uxad027_suppl_supplementary_figure_s4.jpeg]
